# UniMóvil: A Mobile Health Clinic Providing Primary Care to the Colonias of the Rio Grande Valley, South Texas

**DOI:** 10.3389/fpubh.2019.00215

**Published:** 2019-08-21

**Authors:** Eron G. Manusov, Vincent P. Diego, Jacob Smith, Jesús R. Garza, John Lowdermilk, John Blangero, Sarah Williams-Blangero, Francisco Fernandez

**Affiliations:** ^1^Department of Family and Community Medicine, Department of Human Genetics, School of Medicine, University of Texas Rio Grande Valley, Harlingen, TX, United States; ^2^Department of Human Genetics, School of Medicine, South Texas Diabetes and Obesity Institute, The University of Texas Rio Grande Valley, Brownsville, TX, United States; ^3^University of Texas Rio Grande Valley School of Medicine, Edinburg, TX, United States; ^4^VIDAS [United Health Foundation], University of Texas Rio Grande Valley School of Medicine, Edinburg, TX, United States; ^5^Human Development & School Services, University of Texas Rio Grande Valley, Edinburg, TX, United States; ^6^Department of Human Genetics, Genomics Computing Center, School of Medicine, South Texas Diabetes and Obesity Institute, University of Texas Rio Grande Valley, Brownsville, TX, United States; ^7^Department of Human Genetics, School of Medicine, South Texas Diabetes and Obesity Institute, University of Texas Rio Grande Valley, Brownsville, TX, United States; ^8^Department of Psychiatry, School of Medicine, University of Texas Rio Grande Valley, Harlingen, TX, United States

**Keywords:** Colonia, mobile clinic, quality of life, interprofessional care, border, Mexican-American

## Abstract

**Background:** We describe a mobile unit (*UniMóvil*) designed to improve poor healthcare access delivery to residents in two South Texas underserved Colonias. The interprofessional team measured seven clinical outcomes [obesity, diabetes, hypertension, hypertriglyceridemia, low high-density lipoprotein cholesterol (HDL-C) levels, and depression], and using the Duke Health Profile, assessed the health-related quality of life (HrQoL).

**Methods:** The investigators used previously reported disease prevalence, an implementation model, and community needs-assessments to design an outreach healthcare delivery model. A retrospective review of the cohort provides data used to determine potential predictors of clinical variables, 11 domains of HrQOL, and inter/intra Colonia differences.

**Results:** The average age of patients was 45 years-old and females represented 67% of the population served. Results include a high prevalence of obesity (55.5%), hypertension (39%), diabetes (32.5%), and depression (19%), gender differences, and inter-Colonia differences. A generalized linear mixed model analysis provided associations between clinical outcomes and predictors (age, sex, BMI, PHQ-9 score, HbA1c, blood pressure, serum cholesterol, low HDL, triglycerides, and HrQOL domains). The HrQol domain of low self-perceived health, relates to obesity, diabetes, low HDL, and depression. Depression predicted all 11 domains of the HrQol.

**Conclusion:** The prevalence of diabetes, hypertension, obesity, and depression remains epidemic. Mobile clinics increase access and address highly prevalent illnesses in the Colonias. The data collected can be used to address chronic disease and quality of life, focus care, and direct research in high-need underserved areas.

## Introduction

**Figure 1:**
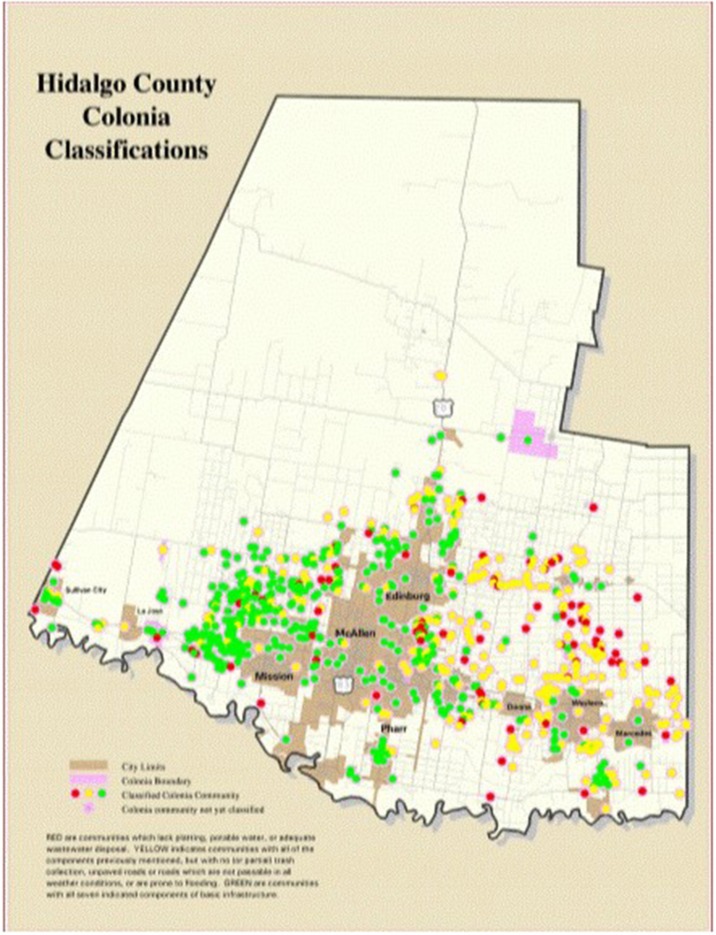
Colonia map in Hidalgo County. Benefit Colonias (3).

Three of the poorest counties in the United States comprise the Rio Grande Valley (RGV) of South Texas; the 38 county area that encompasses the Texas-Mexico Border (1). One in three people in the RGV are uninsured, while 40% of families in the region live on an average of <10,000 dollars per year, and many live in neighborhoods called Colonias (1, 2). The Colonia is defined by the Texas Secretary of State as an economically distressed area that lacks the basic living necessities, such as potable water and sewer systems, electricity, paved roads, and safe and sanitary housing. While frequently found far from resources in unincorporated or rural areas of the counties, a few Colonias are found within city limits (Figure 1) (1, 3).

Dating back to the 1950s, developers in the RGV used agriculturally worthless land, land in flood plains, or rural land, to create un-incorporated subdivisions that lacked basic infrastructure. Families paid low down payments and rent, then built homes with what they could afford, often without indoor plumbing or electricity (1, 4). Some Colonias grew and county commissioners and cities garnered money and services to build basic necessities. Other Colonias continue without county or city services and public health challenges, such as vector borne disease, flooding, and garbage build-up.

The health status of Colonias are characterized as “green” (Colonias that are at low public health risk), “yellow” (Colonias that are at intermediate health risk with potable water and adequate wastewater disposal, but without road paving, drainage, or solid waste disposal), and “red” (Colonias that are at high public and health risk, lacking platting, potable water, and adequate wastewater disposal). As of 2014, 412 “yellow” and 242 “red” Colonias represent nearly half of all Colonias in the RGV (1). Although few studies examine the health of individuals in Colonias along the Texas-Mexico border, those that have, report poor healthcare access, high rates of diabetes (6.8–15.4%), obesity (41.3%), hypertension (5.6–23.6%), hypercholesterolemia (20.4%), and depression (20.4–29%) (5–8). The Colonias that have been incorporated into the city and share the amenities of the city are often classified as green. In contrast, the rural Colonias are often islands of poverty separated by small roads (9, 10). In Colonias geographically separated from critical healthcare resources, the cost of transportation and lack of family vehicles, fear of deportation, low socio-economic and education levels, and chronic illness remains a frequent challenge. Colonias require increased healthcare access, but cost-effective, sustainable care and resources targeted at highly prevalent chronic illness remains under-developed.

The VIDAS *(Valley Interprofessional Dedicated Access and Service)* program supports the development and implementation of primary care services to residents in the Colonias. The clinical arm of VIDAS, the South Texas Interprofessional Teach Collaborative for Health (STITCH) serves to unite the services in the region by building a consortium of professionals to create a model of healthcare for the most vulnerable members of the community. The team met to develop a model that includes a mobile medical unit (UniMóvil), an interprofessional team, and a model of implementation.

The UniMóvil increases access to health services by addressing challenges of transportation common to the Colonias, overcoming language barriers through the inclusion of Spanish speaking healthcare providers, improving health literacy and the use of existing resources, developing trust among new immigrants, and creating a model of continuity-primary care in underserved areas.

The UniMóvil's integrated inter-professional team includes physicians, advanced practice providers, medical assistants, nurses, licensed social workers, pharmacists (PharmD), clinical psychologists, and “Promotoras de Salud,” or community health workers. The team provides primary care services, mental health evaluations and treatment, social services, medication advice and reconciliation, and health and quality-of-life education to Colonia residents. Learners from the University of Texas Rio Grande Valley (UTRGV) and the UTRGV School of Medicine gain experience in integrated interprofessional team-based care and training for future employment. Community organizations, such as the Methodist Healthcare Ministries Wesley Nurses, Unidos Contra Diabetes, Dentists Who Care, local medical centers, and Federally Qualified Health Centers integrate into UniMóvil services and address care-coordination, community capacity building and workforce development.

We present demographic characteristics of a self-selected cross-sectional cohort and report on the services provided and clinical and health related quality of life (HrQol) data. We describe the use of integrated interprofessional teams that serve as learning venues for students and faculty and report on baseline data that confirm the epidemic prevalence of chronic disease in underserved areas. The results can be used to design future research, healthcare access, and services for the Coloniason the US-Mexico border.

## Methods

The protocol was approved by the University of Texas Institutional Review Board. All patients signed a consent for care.

### Clinical Care

The team focused on screening for chronic illness, providing preventive care, and improving health education and quality of life in the region. A stepped wedge-cluster model allowed for progressive implementation into the two communities. The UniMóvil was sequentially trialed in two Colonias at different points on the economic spectrum. Cameron Park was selected as the representative “green” Colonia because of a 15 year history of an established indigent care model and proximity to the Mexico border that facilitates ready access to affordable healthcare in Mexico (6, 7, 11). Indian Hills, a “yellow” Colonia, chosen because of the location in a healthcare access area watershed, low social capital, low socio-economic reserve, and poor transportation resources, represents an earlier stage of development and greater geographical isolation.

The STITCH consortium met to develop a logic model, a method of implementation and evidence-based interventions to address potential issues in the Colonias. County Commissioners met with the group to advise on the needs of the neighborhoods and serve as a conduit to the Colonia residents. A list of possible recommendations, based on previous reports, was used as a starting point for discussion (12). Colonia focus groups were conducted to determine healthcare screening needs, topics of healthcare education interest, and optimal modes of healthcare delivery. Approximately 30 members (primarily women) met in each local Colonia church. The list of identified needs created by the focus groups included: primary care services directed at highly prevalent chronic diseases (hypertension, diabetes, obesity, hyperlipidemia, and depression) as well as school physical exams, immunizations, and topics on preventive medicine.

The following measures were used for screening patients: body mass index (BMI), blood pressure, glycated hemoglobin (HbA1c), total cholesterol, LDL-cholesterol, triglycerides, high density lipoprotein (HDL), and Patient Health Questionnaire-9 (PHQ-9) scores (13–15). Health related Quality of Life (HrQOL) was measured using the Duke Health Profile (Cronbach's alpha 0.55 to 0.78), a tool used in multiple populations (16–21). Duke Health Profile scores demonstrate self-reported functional health status across 11 physical, social, and mental domains (22, 23). The team used Duke Health Profile results to guide the interprofessional team in managing care, to determine differences between Colonias, and to evaluate the relationship between HrQOL and chronic disease in the cohort.

### Description of the Integrated Interprofessional Care Provided

The UniMóvil and a facility located in each Colonia served as a clinic. Prior to and after each session, the team met in a huddle to plan, implement, and evaluate care. Providers (nurse practitioners, physician assistants, family physician faculty and residents in training, PharmD providers, and other learners) offered continuity-primary care with a focus on healthcare maintenance. The interprofessional teams developed healthcare plans that used clinical chemistry results, PHQ9, and Duke Health Profile results. All patients received copies of their laboratory reports and HIPPA compliant follow-up, records, and charts were maintained centrally. At Cameron Park, Methodist Health Ministries Wesley nurses and the Cameron County Proyecto Juan Diego, a not-for-profit community center that has been serving the Brownsville area since 2003 for nutrition and diabetes education, served as referral resources. There were fewer Indian Hills Colonia referral options and were located at least a 30 min drive from the Colonia. Patients who needed more extensive care were referred to local federally qualified health centers and the UTRGV School of Medicine Graduate Medical Education clinics. Health education, counseling, and screening was provided onsite by nurses, community health workers, social workers, and advanced practice providers. The College of Education provided education and health literacy classes. The Promotoras de Salud, instrumental in interfacing between their communities and the UniMóvil, provided basic diabetes care, depression recognition, health education, and oral health screening.

### Analysis: Missing Data Imputation

Although over 1,400 patients were cared for, data were missing for up to 20% of patients. We used a missing data imputation approach that uses a “random forest” algorithm implemented in R (Version 3.2.3) in the MissForest package (24–30). Consequent to imputation using MissForest we arrived at a final data set of 894 observations for all variables of interest.

### Analysis: Logistic and Linear Regression Analysis

Logistic regression models explain odds ratios in the face of more than one variable. We carried out a two-stage regression analysis (logistic or linear depending on the dependent variable). In the first stage, we used the Bayesian Model Averaging (BMA) package in R to carry out Bayesian model selection (BMS) to select the best predictive overall model (31). In the second stage, we performed generalized linear mixed model analysis using the glmm package in R to enable hypothesis testing (i.e., significance testing of regression coefficients) under the BMS selected models, and to allow for potential non-independence within Colonias modeled as a random variable (32).

## Results

The total sex-specific mean and prevalence data for clinical outcomes, PHQ9, and HrQol data are listed in Table 1. Women represented 69.6% of the cohort, with an average age of 45.3 years and a mean BMI of 31.7. The BMI of men averaged 30.81 and the average age was 44.9 years. However, 53% of men and 57% of women recorded a BMI > 30. Equal numbers of women (31%) and men (32%) recorded a HbA1 > 6.5. Males (46%) were more likely to have hypertension (*p* < 0.001), hypertriglyceridemia (60%) (*p* < 0.001), and low HDL (33%) (*p* < 0.001) than females. Of the population screened, men (17%) and women (19%) scored a PHQ9 > 10.

**Table 1:** Sex-specific means for all traits and PHQ9 data.

**Trait**	**Males (*****N*** **= 272)**		**Females (*****N*** **= 622)**		*p*-value^*^
	**Mean**	**S.D**.	**Mean**	**S.D**.	
Age	44.94	13.21	45.33	13.03	0.34
BMI	30.82	5.94	31.74	6.79	0.03
Sys BP	138.5	20.86	132.82	22.74	0.00
Dia BP	83.36	11.47	78.85	11.13	0.00
HbA1C	6.54	1.75	6.47	1.7	0.27
HDL	44.15	9.66	47.78	10.47	0.00
Triglycerides	241.55	118.55	208.48	106.03	0.00
Cholesterol	189.02	34.91	185.87	37.06	0.12
PHQ9	4.87	5.22	5.69	4.96	0.01
Physical health	65.89	22.63	60.93	21.55	0.01
Mental health	76.87	19.24	71.72	17.62	0.00
Social health	66.85	17.03	68.8	17.42	0.06
General health	70.11	15.6	67.04	14.26	0.00
Self-perceived health	71.81	27.26	65.65	28.58	0.00
Self esteem	78.86	15.98	78.33	16.02	0.33
Anxiety	31.58	18.58	33.76	16.53	0.04
Depression	29.75	20.98	34.76	18.98	0.00
Anxiety-depression	26.83	19.19	31.23	17.15	0.00

* *Unpaired sample t-test for a one-tailed hypothesis. S.D., Standard Deviation. Mean differences across males and females was tested by an unpaired t-test assuming equal variance for a one-tailed test*.

Women scored significantly poorer on the Duke Physical Health composite scores (*p* = 0.01) as well as had significant differences on the Duke Mental Health Scores (*p* = 0.00), Duke General Health Scores (*p* = 0.00), Self-Perceived Health Scores (*p* = 0.00), Duke Pain Scores (*p* = 0.01, Duke Anxiety scores (*p* = 0.04), Duke Depression Scores (*p* = 0.00), and the Duke Anxiety-Depression Scores (*p* = 0.00).

Associations between clinical outcomes and predictors (age, sex, BMI, PHQ-9 score, HbA1c, blood pressure, serum cholesterol, low HDL, triglycerides, and HrQOL domains) are listed in Table 2. Obesity hypertension, lipids, age and sex are all related, consistent with known associations. The low Self-perceived Health domain is highly related to obesity, diabetes, low HDL and depression.

**Table 2:** Significant predictors of clinical outcomes based on results of generalized linear mixed models.

**Clinical Outcome *N* = 894**	**Predictor**	**Regression coefficient (S.E.)**	***p*-value**	**Odds ratio**
**Obesity**				
	Sys BP	0.01(0.005)	0.03	1.01
	Dia BP	0.03(0.009)	0.01	1.03
	HDL	−0.04(0.007)	0.00	0.96
	Duke Perceived	−0.01(0.003)	0.00	0.99
**Diabetes**				
	Age	0.05(0.01)	0.00	1.05
	BMI	0.04(0.01)	0.00	1.04
	Triglycerides	0.006(0.001)	0.00	1.006
	Duke Perceived	−0.03(0.003)	0.00	0.974
**Hypertension**				
	Age	0.06(0.007)	0.00	1.06
	BMI	0.08(0.012)	0.00	1.08
	Cholesterol	0.01(0.002)	0.00	1.01
**Hypercholesterolemia**				
	Dia BP	0.05(0.013)	0.00	1.05
	HDL	0.11(0.017)	0.00	1.11
	Triglycerides	0.01(0.001)	0.00	1.01
**Hypertriglyceridemia**				
	HDL	−0.1(0.01)	0.00	0.9
	Cholesterol	0.038(0.003)	0.00	1.04
	HgA1c	0.22(0.06)	0.00	1.25
**Low HDL**				
	BMI	0.05(0.013)	0.00	1.05
	Triglycerides	0.01(0.001)	0.00	1.01
	Cholesterol	−0.03(0.003)	0.00	0.97
	Duke perceived	−0.01(0.003)	0.00	0.99
	Duke anxiety	−0.01(0.006)	0.00	0.99
**Depression**				
	Duke general	−0.08(0.015)	1.60E-07	0.93
	Duke self-perceived	−0.01(0.004)	1.03E-10	0.99
	Duke Depression	0.07(0.011)	1.64E-24	1.07

Predictors of the 11 Duke Domains are listed in Table 3. Although each clinical measure was an important predictor of a domain of HrQOL, the most consistent predictors, PHQ9 score, age, sex, weight, and diabetes reduced HrQol in all patients. Mean scores on the 11 domains of the Duke Profile are listed in Table 4. Indian Hills Colonia residents ranked higher in perceived health.

**Table 3:** The 11 Duke Health Profiles linear mixed model results: predictors of 11 Duke Health Profiles from linear mixed model results.

**Trait**	**Parameter**	**Estimate**	**S.E**.	***p*-value**
**Physical**				
	β Age	−0.286	0.044	0.00
	β BMI	−0.585	0.088	0.00
	β Tg	0.015	0.005	0.00589
	β PHQ9	−2.442	0.113	0.00
**Mental health**				
	β Sex	−3.346	1.016	0.00
	β PHQ9	−2.288	0.093	0.00
**Social health**				
	β Age	0.124	0.042	0.01
	β PHQ9	−1.251	0.107	0.00
**General health**				
	β PHQ9	−1.973	0.072	0.00
**Perceived health**				
	β Sex	−4.813	1.823	0.01
	β BMI	−0.463	0.128	0.00
	β Chol	0.094	0.023	0.00
	β PHQ9	−1.861	0.167	0.01
	β HgA1C	−4.588	0.5	0.00
**Self esteem**				
	β Age	0.118	0.037	0.00
	β PHQ9	−1.406	0.095	0.00
**Anxiety score**				
	β PHQ9	2.111	0.089	0.00
**Depression score**				
	β Sex	2.773	1.033	0.00
	β PHQ9	2.679	0.094	0.00
**Anxiety-depression**				
	β Sex	2.385	0.945	0.00
	β PHQ9	2.411	0.086	0.00
**Pain score**				
	β Age	0.288	0.069	0.00
	β PHQ9	2.259	0.178	0.00
**Disability score**				
	β systolic BP	0.068	0.029	0.02
	β PHQ9	1.176	0.13	0.00

*β is the regression coefficient*.

**Table 4:** Duke Health Profile for Cameron Park and Indian Hills Colonias.

**Domain**	**Cameron Park**			**Indian Hills**			
	***N***	**Mean**	**S.D**.	***N***	**Mean**	**S.D**.	*p*-value^*^
Physical	330	62.64	23.69	325	63.74	20.13	0.26
Mental health	330	74.66	19.41	325	74.01	16.78	0.32
Social score	330	69.26	17.1	325	67.27	16.03	0.06
General score	330	68.69	15.87	325	68.45	13.19	0.42
Perceived health	330	64.83	29.6	325	69.46	25.4	0.02^*^
Self esteem	330	79.21	15.96	325	78.71	14.57	0.34
Anxiety score	330	31.76	18.48	325	33.05	15.05	0.16
Depression score	330	32.14	21.12	325	32.43	17.93	0.43
Anxiety depression	330	28.45	19.35	325	29.27	15.76	0.28
Pain score	330	45.8	32.11	325	45.17	27.05	0.39
Disability	330	8.29	20.78	325	7.82	20.38	0.38

* *Unpaired sample t-test for a one-tailed hypothesis significant at P = 0.02*.

## Discussion

The VIDAS/STITCH project increased access to healthcare for residents in two Colonias, provided a learning ground for interprofessional education, and served as an impetus for a more extensive development of healthcare delivery in the region. Although the prevalence of obesity as a national trend is slowing, the prevalence of obesity remains disproportionately high in Hispanics, and especially in Mexicans and Mexican Americans living on the US-Mexico border (1, 2). Obesity, diabetes, hypertension, hypertriglyceridemia, low HDL, and depression were highly prevalent in our cohort. Males had a higher prevalence of hypertension, hypertriglyceridemia and lower HDL than females. The reason for these sex differences, however, are unclear. Since many men in the Colonias work until late in the evening and on weekends, they are less likely to seek primary care services. It is also possible that the males who seek care are more than likely unable to work due to illness or injury and thus have worse health profiles. Alternatively, there may be socio-cultural reasons why the Hispanic males living in the Colonias do not seek healthcare. To address healthcare needs for all residents, it is important to determine ways to increase access for males. We tried evening/weekend events and increased recruitment efforts for males without success.

The results support other well-known association between age, obesity, hypertension, hypercholesterolemia, hypertriglyceridemia, low HDL levels, and depression that we have considered in iterative changes to the implementation and evaluation of our model. We added more resources for nutrition education and mental health services. We screened all patients, learners, providers and mental health specialists trained in screening, brief intervention and referral for treatment (SBIRT). Social determinants of health, including job insecurity, poverty, lack of access to care, transportation, family violence, substance use, diet, language challenges, lack of education, immigrant status, acculturation and lack of trust may contribute to resident's depression and subsequently to HrQoL. In subsequent years, we added a screen for Social Determinants of Health, although significant changes will require more targeted research, new policies, laws, and the involvement of government resources. Our patients scored high on the PHQ9 screen related to all domains of HrQol and chronic illness. More research and implementation of programs to address depression and the root causes of depression are needed. It is possible that focusing on addressing the symptoms of depression may improve health related quality of life and possibly chronic disease.

Chronic illness affects the domains of functional, physical, and social quality of life, while age, sex and BMI remain important predicators of HrQOL. The Duke Profile domain for perceived health is a separate one-item indicator of the extent to which the respondent judges herself or himself to be “basically” healthy. Poor self-perceived health is a measure of quality of life and is related to education, language differences, age, and mental illness (16, 19, 33). Our cohort did not perceive their health as poor, despite the prevalence of chronic illness and other risk factors. Further research using sociocultural or social cognitive theories could further explain the reasons for this. It is possible that un-measured social determinants of health, poverty, lack of access to health care, language and cultural differences, as well as immigration status contribute to the level of self-perceived health in the Colonias. The exact correlation is unknown.

Based on the results of the multivariate tests for CP and IH, the Colonias do not report differences in 10 of the 11 Duke Profile domains. Further research can examine why the Colonia with less access to medical care, closer proximity to the border, and increased isolation reported a higher self-perceived level of health. Possible explanations include sampling errors (Berkson's Paradox: patients are not typical in the treatment group), immigration paradox (new immigrants are healthier or perceive themselves to be healthier) or other explanations such as socio-economic, cultural, or health related contributors to perceived health.

## Implication for Practice

The Colonia Care Project and the provision of integrated, interprofessional, primary care by teams of providers that plan, deliver, and evaluate together is a model of care that can be effective in medically underserved areas across the US. The impact of integrated interprofessional care may be seen in regions where access to care, difficulty with transportation, and poverty affect healthcare delivery.

The healthcare challenges associated with immigrant populations, poverty, transportation difficulties, and poorly developed communication technology infrastructure can magnify challenges living on the border. To achieve health equity and to improve access to healthcare in a vulnerable population, this project focused on the integration of care, preventive care, primary care, and healthcare education. Our initial outcomes suggest that not only is it important to integrate care at the level of the community with needs assessment, building trust, and providing culturally appropriate care, but that a focus on health-related quality of life may be as important for improving HrQol. Evidence-based research is necessary to determine what clinical services should be provided in un-insured and underserved populations in the Colonias, the relationship between health equity and HrQoL/chronic disease, and how we use integrated interprofessional resources to best serve our communities.

## Implications for Research

Although successful in many aspects, our experience suggests important opportunities for clinical implementation, as well as evidence-based research design. Our self-selected cohort may represent a group that developed a trusting relationship or took advantage of resources that other Colonia residents did not take. The design of prospective studies on gender differences in healthcare and health, on why Hispanic males may not readily seek healthcare services, and if there are specific fears or services that are also gender specific (HIV testing, PSA testing, screening for colon cancer, and mental health/substance abuse) are needed. Implementation science with prospective measures and group comparisons can provide better data to support clinical approaches and to determine possible differences within and between Colonias. Research opportunities include genomic research to identify underlying risk factors for comorbid depression, as well as obesity, diabetes, liver disease, cancer, and hypertension. This type of collaborative effort could both generate a focused integrated healthcare delivery and simultaneously identify endophenotypic markers that will index genetic risk. This ultimately will allow us to develop an innovative prevention strategy based on state-of-the art multi-omic approaches.

## Conclusion

This project reports a community-driven, interprofessional collaboration aimed at increasing access to primary care in a medically isolated population on the Texas-Mexico border. The project was successful in providing care to underserved, uninsured residents in the Colonias of South Texas. The project developed a medical mobile clinic, community capacity building/workforce development with community healthcare workers and learners from the local universities, and a model of care that incorporates existing systems and organizations. The report is unique in that it analyzes clinical data collected from residents that live in areas of poverty, linguistic and cultural variation, and with a threatened immigration status. Residents suffer from disproportionately high rates of diabetes, obesity, hypertension, and depression. The role of environmental health threats, genetic influences, and differential exposure to the social determinants of health are yet to be studied extensively. Further studies are needed to elucidate the role of health, genetic, environmental, economic and social factors on health, the quality of life, and depression.

## Author Contributions

All authors listed have made a substantial, direct and intellectual contribution to the work, and approved it for publication.

### Conflict of Interest Statement

The authors declare that the research was conducted in the absence of any commercial or financial relationships that could be construed as a potential conflict of interest.
